# Isoliensinine induces apoptosis in triple-negative human breast cancer cells through ROS generation and p38 MAPK/JNK activation

**DOI:** 10.1038/srep12579

**Published:** 2015-07-29

**Authors:** Xiyu Zhang, Xiyao Wang, Tingting Wu, Boxuan Li, Tianqi Liu, Rong Wang, Qiao Liu, Zhaojian Liu, Yaoqin Gong, Changshun Shao

**Affiliations:** 1 Key Laboratory of Experimental Teratology, Ministry of Education and Department of Molecular Medicine and Genetics, Shandong University School of Medicine, Jinan, Shandong 250012, China; 2 Department of Physiology, Shandong University School of Medicine, Jinan, Shandong 250012, China; 3 Department of Cell Biology, Shandong University School of Medicine, Jinan, Shandong 250012, China

## Abstract

Isoliensinine, liensinine and neferine are major bisbenzylisoquinoline alkaloids in the seed embryo of lotus (*Nelumbo nucifera)*, and exhibit potential anti-cancer activity. Here, we explored the effects of these alkaloids on triple-negative breast cancer cells and found that among the three alkaloids isoliensinine possesses the most potent cytotoxic effect, primarily by inducing apoptosis. Interestingly, isoliensinine showed a much lower cytotoxicity against MCF-10A, a normal human breast epithelial cell line. Further studies showed that isoliensinine could significantly increase the production of reactive oxygen species (ROS) in triple-negative breast cancer cells, but not in MCF-10A cells. The isoliensinine-induced apoptosis could be attenuated by radical oxygen scavenger N-acetyl cysteine, suggesting that the cytotoxic effect of isoliensinine on cancer cells is at least partially achieved by inducing oxidative stress. We found that both p38 MAPK and JNK signaling pathways were activated by isoliensinine treatment and contributed to the induction of apoptosis. Furthermore, inhibitors or specific siRNAs of p38 MAPK and JNK could attenuate apoptosis induced by isoliensinine. However, only the p38 inhibitor or p38-specific siRNA blocked the elevation of ROS in isoliensinine-treated cells. Our findings thus revealed a novel antitumor effect of isoliensinine on breast cancer cells and may have therapeutic implications.

Breast cancer is the most common cancer among women. Triple-negative breast cancers (TNBCs), lacking estrogen receptor (ER), progesterone receptor, and ERBB2 (also known as HER-2/neu), are more aggressive and difficult to treat than other subtypes of breast cancers. TNBC accounts for 12 to 17% of breast cancer cases^1^. Although approximately 50% of patients with TNBC respond to conventional chemotherapies, including taxanes, anthracyclines, cyclophosphamide, and platinum salts^2^, these treatments are associated with serious side effects. Therefore, novel agents need to be developed for TNBC patients.

Natural compounds are a valuable source for new drug development. Some alkaloids isolated from plants have already been successfully developed into chemotherapeutic drugs, such as camptothecin (CPT)^3^ and vinblastine^4^. Isoliensinine and its analogues liensinine and neferine are the major bisbenzylisoquinoline alkaloids extracted from the seed embryo of lotus (*Nelumbo nucifera* Gaertn). In a previous study, we found that neferine inhibited proliferation of human osteosarcoma cells by inducing G1 cell cycle arrest^5^. Neferine was also shown to induce ROS generation leading to apoptosis in HepG2 cells^6^. Additionally, neferine could reverse multidrug resistance of human gastric carcinoma cells^7^. Recently, isoliensinine and liensinine were also shown to exhibit potent cytotoxic effect towards cancer cells (including MCF-7 cells) and function as an enhancer of autophagy in apoptosis-defective cells^8^.

Oxidative stress is involved in a number of physiological and pathological processes, including cancer, neurodegenerative diseases, and arteriosclerosis. Reactive oxygen species are products of normal metabolism and xenobiotic exposure, and depending on their concentration, ROS can be beneficial or harmful to cells and tissues^9^. Accumulating evidence has suggested that cancer cells have higher ROS levels than normal cells and are more vulnerable when encountering further ROS insults induced by exogenous agents^10^. Excessive ROS can induce cell death including apoptosis, autophagy and necrosis^11^,^12^. Several studies have demonstrated that apoptotic cell death induced by ROS is mediated by p38 MAPK and JNK activation^13^,^14^,^15^.

Therefore, in the present study we assessed anti-cancer effects of isoliensinine, liensinine and neferine on triple-negative human breast cancer cells. Our data indicated that isoliensinine possesses the most potent anti-cancer activity among the three alkaloids. The level of apoptosis was significantly elevated in cancer cells treated with isoliensinine. Importantly, we demonstrated that the pro-apoptotic effect of isoliensinine was mediated by an increase in ROS production and the activation of p38 MAPK and JNK pathways.

## Results

### Isoliensinine selectively inhibits proliferation and colony formation of human breast cancer cells

**Figure 1:**
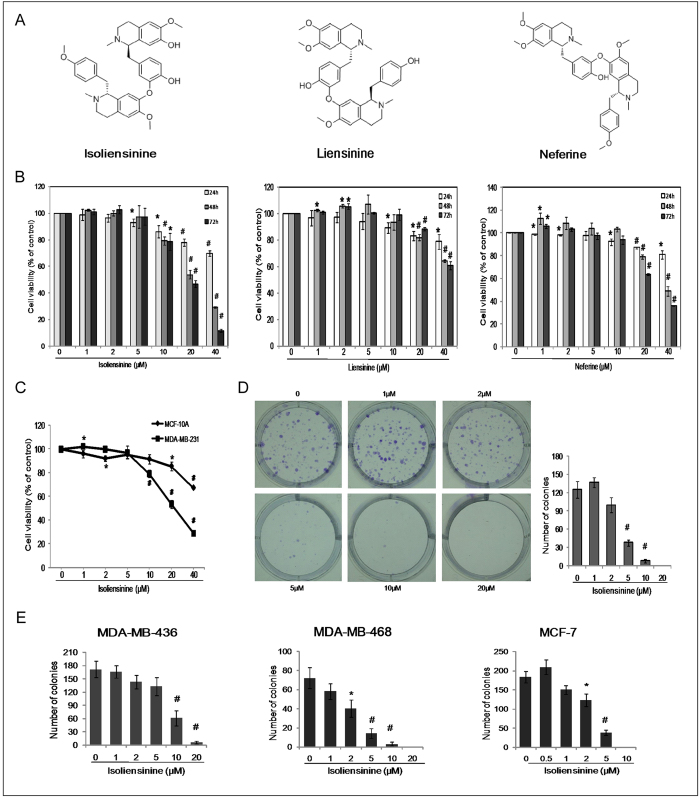
Effect of isoliensinine on growth in human breast cancer cells and normal human breast epithelial cells. **A**, chemical structures of isoliensinine, liensinine and neferine. **B**, MDA-MB-231 cells were treated with various concentrations of isoliensinine, liensinine or neferine for 24, 48 and 72 h. **C**, MDA-MB-231 and MCF-10A cells were exposed to isoliensinine (1–40 μM) or vehicle control (0.1% DMSO) for 48 h. Cell viability was measured by CCK-8 assay. The experiments were performed in triplicate. Data presented as means ± S.D. are representative of three independent experiments. *P < 0.05, #P < 0.01, when compared with control group. **D**,**E**, the colony formation assay was performed in MDA-MB-231, MDA-MB-436, MDA-MB-468 and MCF-7 cells. Breast cancer cells were plated on 6-well plate at a density of 1 × 10^3^ cells/well and treated with isoliensininie (1–20 μM) for 48 h. Media were changed after 48 h of incubation. Colonies were observed until 2 weeks. The data shown here are from a representative experiment repeated three times with similar results. Data are presented as means ± S.D. *P < 0.05, #P < 0.01, when compared with control group.

Isoliensinine, liensinine and neferine are major bisbenzylisoquinoline alkaloids extracted from the seed embryo of *Nelumbo nucifera* Gaertn and their structures are depicted in Fig. 1A. These alkaloids were previously shown to have potent cytotoxic effects on some human cancer cell lines^5^,^6^,^8^. We first investigated the inhibitory effects of isoliensinine, liensinine and neferine on the growth of human breast cancer cell line MDA-MB-231. MDA-MB-231 cells were treated with 1–40 μM isoliensinine, liensinine and neferine for 24, 48 and 72 h and were then subjected to cell viability assay. We observed that isoliensinine was most potent among the three alkaloids, while liensinine was the least toxic (Fig. 1B). Therefore, we focused on the anti-cancer property of isoliensinine in MDA-MB-231cells. The IC_50_ values of isoliensinine were estimated to be 108.1 μM (24 h), 22.78 μM (48 h) and 18.34 μM (72 h) respectively.

Next, we examined the effect of isoliensinine on the growth of normal human breast epithelial cells MCF-10A and found that MCF-10A cells were less sensitive to isoliensinine than the MDA-MB-231 cells (Fig. 1C). The IC_50_ values were estimated to be 151 μM (24 h), 86.22 μM (48 h) and 63.89 μM (72 h) for MCF-10A cells (Fig. S1), while IC_50_ at 48 h was nearly four-fold higher than that for MDA-MB-231 cells. The anti-proliferation effect of isoliensinine on MDA-MB-231 cells was confirmed by colony formation assay. As shown in Fig. 1D, isoliensinine treatment for 48 h significantly reduced the number of colonies in a dose-dependent manner when compared with untreated cells. We also determined the anti-proliferation effect of isoliensinine on other human breast cancer cells, including TNBC cells MDA-MB-436 and MDA-MB-468, triple-positive breast cancer cells MCF-7. Isoliensinine treatment similarly markedly inhibited the colony formation of these breast cancer cells (Fig. 1E).

### Isoliensinine induces cell cycle arrest at G1 phase

**Figure 2:**
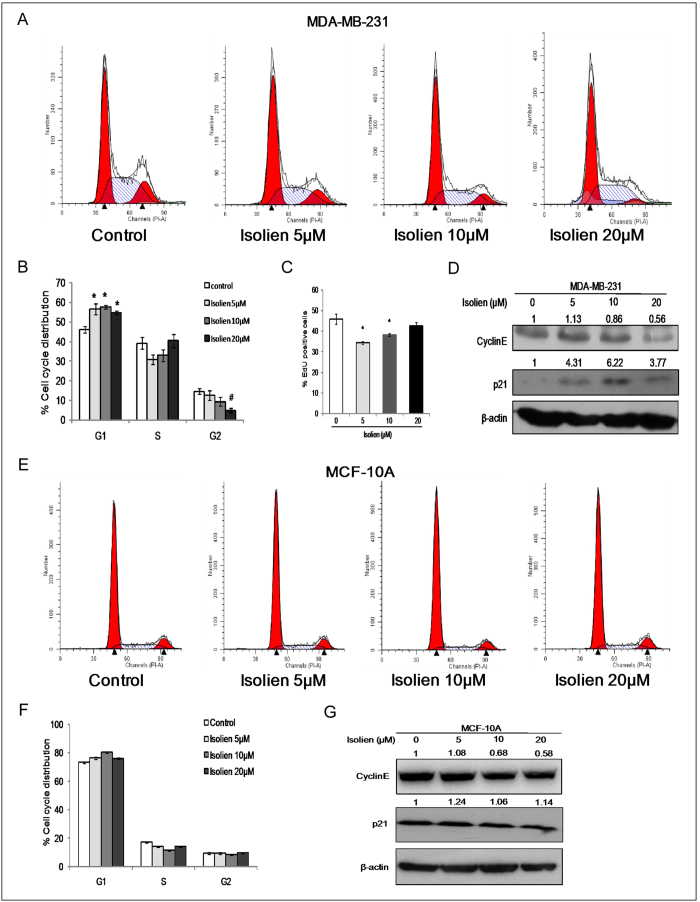
Effect of isoliensinine on cell cycle distribution in MDA-MB-231 cells and normal human breast epithelial cells. **A**, isoliensinine induced G1 cell cycle arrest in MDA-MB-231 cells. MDA-MB-231 cells were treated with 5, 10 and 20 μM isoliensinine for 48 h. 0.1% DMSO was used as control. The distribution of cell cycle was assessed by flow cytometry. **B**, the percentage of cells in each phase is shown as the mean ± S.D. from three independent experiments. *P < 0.05, #P < 0.01, when compared with control group. **C**, EdU proliferation assay was performed 48 h after the incubation with 5–20 μM isoliensinine in MDA-MB-231 cells. Ratio of EdU positive cells is shown as the mean ± S.D. from three independent experiments. *P < 0.05, when compared with control group. **D**, MDA-MB-231 cells were treated with isoliensinine (5–20 μM) for 48 h. Whole cell lysates were analyzed by immunoblotting with antibodies specific for cyclin E and p21. β-actin was used as a loading control. **E**, effect of isoliensinine on cell cycle distribution in MCF-10A cells. MCF-10A cells were incubated with 5–20 μM isoliensinine for 48 h. 0.1% DMSO was used as control. The distribution of cell cycle was assessed by flow cytometry. **F**, the percentage of cells in each phase is shown as the mean ± S.D. from three independent experiments. **G**, MCF-10A cells were exposed to 5–20 μM isoliensinine for 48 h. Whole cell lysates were analyzed by immunoblotting with antibodies specific for cyclin E and p21. β-actin was used as a loading control. All blots were performed under the same experimental conditions. Band intensities were quantified by ImageJ and normalized to β-actin. Data are expressed as a fold change relative to the control. The full-length blots are included in the supplementary information (Figure S3).

To determine whether the growth-inhibitory effect of isoliensinine was mediated by cell cycle arrest, cell cycle distribution in MDA-MB-231 cells was examined by flow cytometry. As shown in Fig. 2A,B, isoliensinine treatment for 48 h led to an increase in the proportion of cells at G1 phase and a reduction at G2 phase. The percentages of cells at G1 phase at 48 h after the incubation with isoliensinine were increased to 56.53% (5 μM), 57.71% (10 μM) and 54.71% (20 μM), from 46.12% in the control. Meanwhile, percentages of cells at G2 phase at 48 h were decreased to 12.57% (5 μM), 9.33% (10 μM) and 4.85% (20 μM), from 14.63% in the control. Interestingly, while isoliensinine at 5 μM resulted in a significant reduction of the cell population at S phase, to 30.9% from 39.25% in the control, such an effect was not detected at 20 μM. EdU incorporation assay confirmed that the percentages of cells at S phase declined in MDA-MB-231 cells treated by isoliensinine(Fig. 2C). To examine the underlying mechanism responsible for isoliensinine-induced G1 arrest, we next analyzed several proteins that are involved in the regulation of G1/S progression in MDA-MB-231 cells by Western blot analysis. The level of cyclin E was markedly decreased by isoliensinine in a dose-dependent manner (Fig. 2D). We previously reported that neferine-induced G1 arrest was mediated by up-regulation of p21 in U2OS cells^5^. Therefore we examined the level of p21 in isoliensinine-treated MDA-MB-231 cells and found that the level of p21 was also increased in MDA-MB-231 cells after exposure to isoliensinine for 48 h (Fig. 2D). These results suggested that isoliensinine may induce G1 cell cycle arrest through downregulation of cyclins and upregulation of p21.

Next, we determined the effect of isoliensinine on cell cycle distribution in MCF-10A cells. Unlike MDA-MB-231 cells, changes in cell cycle distribution were very marginal (Fig. 2E,F). Correspondingly, the level of p21 was not significantly increased (Fig. 2G).

### Isoliensinine induces apoptosis through mitochondrial pathway

**Figure 3:**
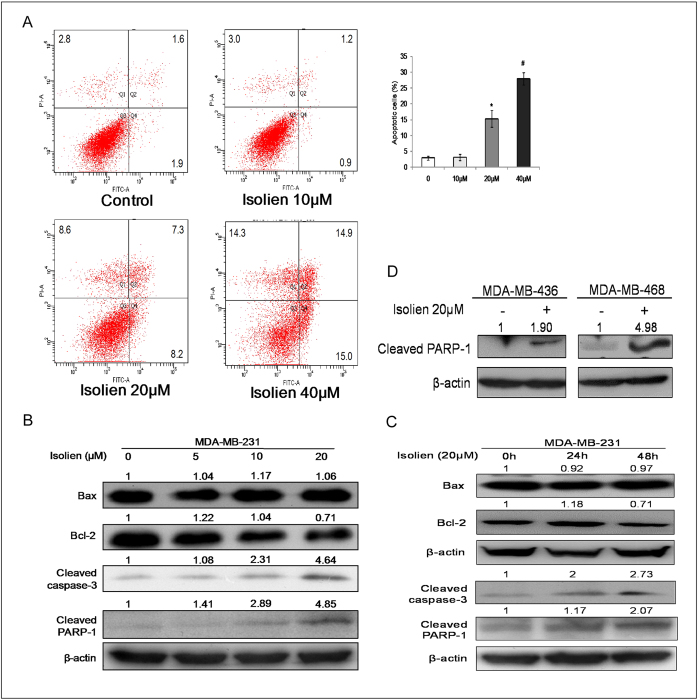
Pro-apoptotic effect of isoliensinine on triple-negative breast cancer cells. **A**, MDA-MB-231 cells were exposed to 10, 20, and 40 μM isoliensinine for 48 h. 0.1% DMSO was used as control. Cells were processed by flow cytometry using Annexin V/PI staining. The percentage of Annexin V-positive population indicates apoptosis induction at every concentration of isoliensinine. Results shown are representative of three independent experiments. *P < 0.05, #P < 0.01, when compared with control group. **B**,**C**, expression modulation of apoptosis-related proteins by isoliensinine in MDA-MB-231 cells. Cells were treated with 5–20 μM isoliensinine for 48 h (**B**, dose-dependent study) and 20 μM isoliensinine for 0, 24 and 48 h (**C**, time-dependent study). Cell lysates were prepared and subjected to western blotting for Bax, Bcl-2, cleaved caspase-3 and PARP-1. **D**, MDA-MB-436 and MDA-MB-468 cells were incubated with 20 μM isoliensinine for 24 h. Cell lysates were prepared and analyzed by western blotting for cleaved PARP-1. β-actin was used as a loading control. Band intensities were quantified by ImageJ and normalized to β-actin. Data are expressed as a fold change relative to the control. The full-length blots are included in the supplementary information (Figure S4 and S5).

We next investigated whether isoliensinine could induce apoptosis in MDA-MB-231 cells. MDA-MB-231 cells were treated with 10–40 μM isoliensinine for 48 h, and then apoptotic cells were determined by annexin V staining. Isoliensinine treatment for 48 h dose-dependently increased the percentages of apoptotic cells, from 3.5% in the control to 15.5% (20 μM) and 29.9% (40 μM) (Fig. 3A). When MDA-MB-231 cells were incubated with 20 μM isoliensinine for 24, 48 and 72 h, respectively, the percentages of apoptotic cells were increased to 17.8% (48 h), to 31.1% (72 h) (Fig. S2A), indicating a time-dependent pro-apoptotic effect of isoliensinine.

An imbalance between pro-apoptotic protein Bax and anti-apoptotic protein Bcl-2 would result in the release of cytochrome C from mitochondria, caspase-3 activation and subsequent apoptosis. Therefore, we next examined the levels of Bax and Bcl-2 by Western blot analysis in isoliensinine-treated cells. MDA-MB-231 cells were treated with isoliensinine (5–20 μM) for 48 h, or were exposed to 20 μM isoliensinine for various lengths of time. We found that isoliensinine down-regulated the expression level of Bcl-2 in a dose-dependent manner, reducing the Bcl-2/ Bax ratio to 0.89 (10 μM), 0.67(20 μM) compared to untreated cells (Fig. 3B).

Activation of caspase-3 and cleavage of PARP-1 are hallmarks of apoptosis. We therefore next examined the levels of cleaved caspase-3 and PARP-1 in MDA-MB-231 cells treated by isoliensinine. As shown in Fig. 3B,C, isoliensinine triggered the cleavage of caspase-3 and PARP-1 in a dose- and time-dependent manner. We also determined the pro-apoptotic effect of isoliensinine on MDA-MB-436 and MDA-MB-468 cells. The cleavage of PARP-1 was markedly increased after cells were exposed to 20 μM isoliensinine for 24 h (Fig. 3D). But we detected no increased apoptosis in isoliensinine-treated MCF-7 cells (Fig. S2B). Together, these data indicate that isoliensinine could efficiently induce apoptosis in triple-negative breast cancer cells.

### Isoliensinine selectively increases ROS levels in cancer cells

**Figure 4:**
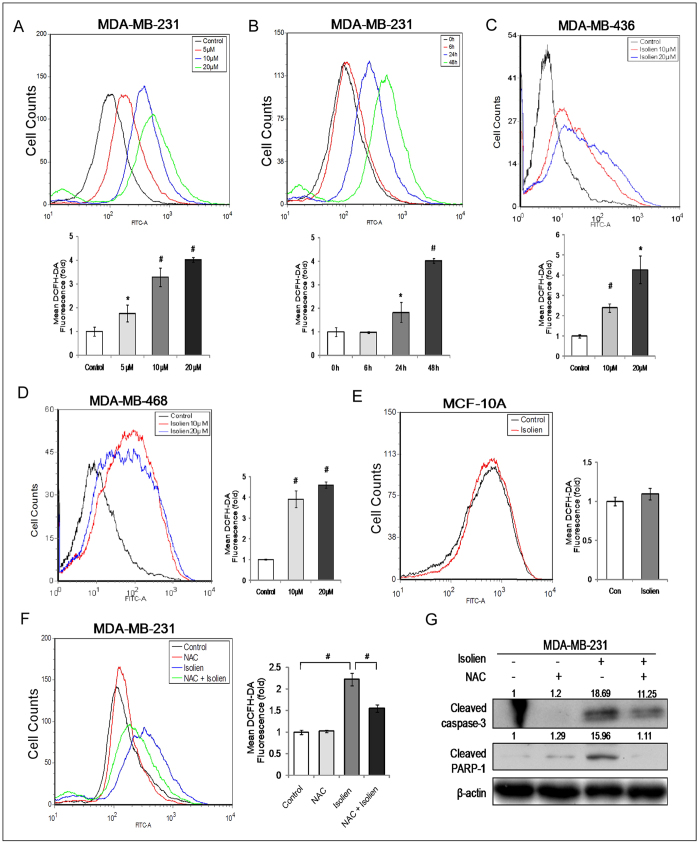
The accumulation of ROS production is required for apoptosis induced by isoliensinine in triple-negative breast cancer cells. ROS generation was measured using oxidation sensitive fluorescent probe (DCFH-DA) by flow cytometry. **A**,**B**, MDA-MB-231 cells were treated with 5–20 μM isoliensinine for 48 h (**A**, dose-dependent study) and 20 μM isoliensinine for 0, 6, 24 and 48 h (**B**, time-dependent study). **C**, MDA-MB-436 cells were incubated with 10–20 μM isoliensinine for 24 h. **D**, MDA-MB-468 cells were treated with 10–20 μM isoliensinine for 24 h. Data presented as means ± S.D. are representative of three independent experiments. *P < 0.05, #P < 0.01, when compared with control group. **E**, isoliensinine did not increase ROS levels in normal cells MCF-10A. MCF-10A cells were treated with 20 μM isoliensinine for 48 h, and ROS levels were mearsured by flow cytometry. Data presented as means ± S.D. are representative of three independent experiments. **F**,**G**, MDA-MB-231 cells were pretreated with or without 10 mM NAC for 1 h, and then followed by 20 μM isoliensinine for 48 h. **F**, isoliensinine induced ROS elevation and NAC could attenuate the increase of ROS levels. Data presented as means ± S.D. are representative of three independent experiments. #P < 0.01, when compared with the indicated group. **G**, effect of isoliensinine on expression of apoptosis-related proteins in the absence or presence of NAC. Western blotting was performed for cleaved caspase-3 and PARP-1. β-actin was used as a loading control. Band intensities were quantified by ImageJ and normalized to β-actin. Data are expressed as a fold change relative to the control. The full-length blots are included in the supplementary information (Figure S6).

Isoliensinine has been shown to possess an anti-oxidant property^16^. However, neferine, an analogue of isoliensinine, has been reported to exhibit either anti-oxidant or pro-oxidant activities^6^,^17^,^18^. To determine whether isoliensinine has any effect on ROS level in MDA-MB-231 cells, we used fluorescent probe (DCFH-DA) to monitor the intracellular ROS level in the presence or absence of isoliensinine. As shown in Fig. 4A, MDA-MB-231 cells that were treated with 5–20 μM isoliensinine for 48 h had significantly higher ROS levels than control cells. ROS levels were found to be increased by 1.76 (5 μM), 3.28 (10 μM) and 4.02-folds (20 μM) by isoliensinine over that in untreated cells. Isoliensinine at 20 μM induced ROS generation in a time-dependent manner, with 1.82 and 4.02-fold increase over untreated control at 24 and 48 h respectively (Fig. 4B). Similar results were obtained in MDA-MB-436 and MDA-MB-468 cells (Fig. 4C,D). These results suggest that isoliensinine may increase ROS production in triple-negative breast cancer cells. In contrast to the remarkable induction of ROS by isoliensinine in MDA-MB-231, MDA-MB-436 and MDA-MB-468 cells, isoliensinine at 20 μM for 48 h did not lead to ROS elevation in MCF-10A cells (Fig. 4E), suggesting that the induction of ROS by isoliensinine could be specific to cancer cells.

To determine whether the isoliensinine-induced apoptosis in triple-negative breast cancer cells is mediated by elevated ROS level, we examined apoptosis induced by isoliensinine in cells pretreated with ROS scavenger N-acetyl cysteine (NAC). MDA-MB-231 cells were pretreated with or without 10 mM NAC for 1 h, and were then subjected to isoliensinine for additional 48 h. As expected, pretreatment of cells with NAC caused a significant drop of ROS level in isoliensinine-treated MDA-MB-231 cells (Fig. 4F). Importantly, blocking ROS generation by NAC led to a remarkable decline in the levels of cleaved-caspase-3 and cleaved PARP-1 (Fig. 4G). These data suggest that isoliensinine-induced apoptosis in triple-negative breast cancer cells is likely to be mediated by increased ROS accumulation. Taken together, these results indicate that isoliensinine could selectively increase ROS accumulation and induce apoptosis in triple-negative breast cancer cells.

### Isoliensinine activates p38 MAPK and JNK pathways

**Figure 5:**
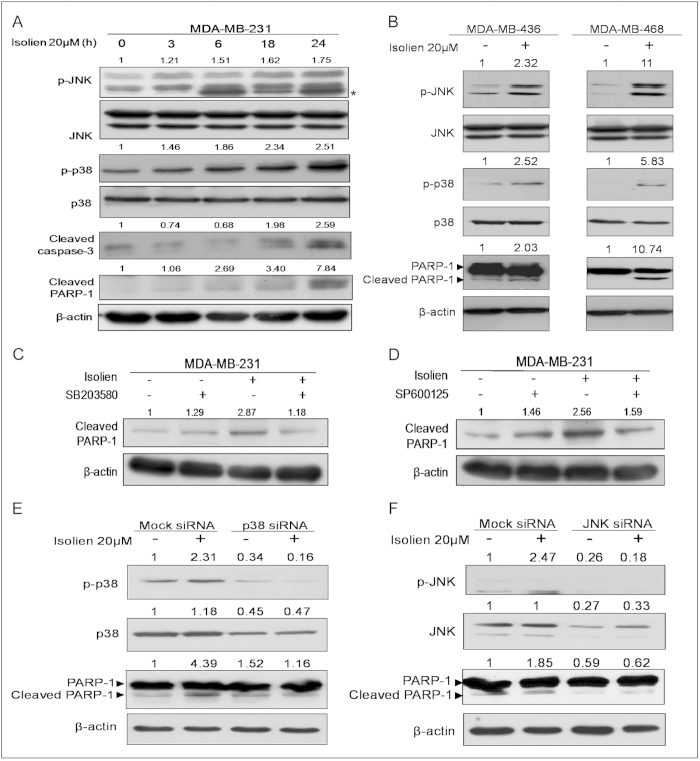
p38 MAPK and JNK pathways mediate isoliensinine-induced apoptosis in triple-negative breast cancer cells. **A**, time course of p38 MAPK and JNK actvation. MDA-MB-231 cells were treated with 20 μM isoliensinine for various lengths of time. **B**, MDA**-**MB-436 and MDA-MB-468 cells were incubated with 20 μM isoliensinine for 24 h. **C**,**D**, MDA-MB-231 cells were treated with 20 μM isoliensinine alone or in combination with 10 μM SB203580 (**C**) or 10 μM SP600125 (**D**) for 24 h. Cell lysates were prepared and analyzed by western blotting for cleaved PARP-1. β-actin was used as a loading control. **E**, p38 siRNA rescued isoliensinine-induced apoptosis. MDA-MB-231 cells were transfected with mock siRNA or p38 siRNA, and incubated with 20 μM isoliensinine for 24 h. **F**, JNK siRNA blocked isoliensinine-induced apoptosis. MDA-MB-231 cells were transfected with mock siRNA or JNK siRNA, and treated with 20 μM isoliensinine for 24 h. Western blot analysis was performed using total cell lysates to examine the phosphorylation levels of p38 and JNK and cleaved caspase-3 and PARP-1 protein levels. β-actin was used as a loading control. For cleaved caspase-3 and cleaved PARP-1, band intensities were quantified by ImageJ and normalized to β-actin. For p-JNK1 and p-p38, band intensities were normalized to JNK1 and p38, respectively. Data are expressed as a fold change relative to the control. Data shown are representative of three independent experiments. The full-length blots are included in the supplementary information (Figure S7, S8 and S9).

Many anticancer compounds induce ROS formation and activate MAPK signaling, and ultimately cause apoptosis in cancer cells^19^,^20^. In our previous study, we have shown that neferine could inhibit proliferation of human osteosarcoma cells and activate p38 MAPK and JNK pathways^5^. We next examined the phosphorylation (activation) status of the p38 MAPK and JNK proteins. MDA-MB-231cells were exposed to 20 μM isoliensinine for various lengths of time (3 to 24 h) and the activations of the p38 MAPK and JNK pathways were evaluated by immunoblotting. The levels of phosphorylated p38 MAPK and JNK were gradually increased after the isoliensinie treatment (Fig. 5A). Meanwhile, the levels of cleaved caspase-3 and PARP-1 were elevated in a time-dependent manner in MDA-MB-231 cells (Fig. 5A). In addition, MDA-MB-436 cells and MDA-MB-468 cells also showed activation of p38 MAPK and JNK in response to 20 μM isoliensinine treatment for 24 h (Fig. 5B). These results suggested that p38 MAPK and JNK pathways might mediate isoliensinine-induced apoptosis.

We then examined whether activation of p38 MAPK and JNK pathways was necessary for isoliensinine-induced apoptosis in MDA-MB-231 cells. Cells were first pretreated with p38 MAPK inhibitor SB203580 and JNK inhibitor SP600125, respectively, before they were treated by isoliensinine for additional 24 h. Notably, SB203580 and SP600125 significantly reduced the levels of PARP-1 cleavage induced by isoliensinine (Fig. 5C,D), indicating that SB203580 and SP600125 could protect MDA-MB-231 cells from isoliensinine-induced apoptosis. To confirm the role of p38 MAPK and JNK activation in isoliensinine-induced apoptosis, we depleted p38 MAPK and JNK, respectively, with siRNAs. As shown in Fig. 5E,F, isoliensinine-induced phosphorylation of p38 MAPK or JNK was subdued in p38 or JNK siRNA transfected cells compared with mock siRNA transfected cells. Meanwhile the levels of cleaved PARP-1 were also reduced. These results suggested that isoliensinine-induced apoptosis in triple-negative breast cancer cells was mediated by p38 MAPK and JNK pathways.

### Role of p38 MAPK and JNK pathways in ROS induction by isoliensinine

**Figure 6:**
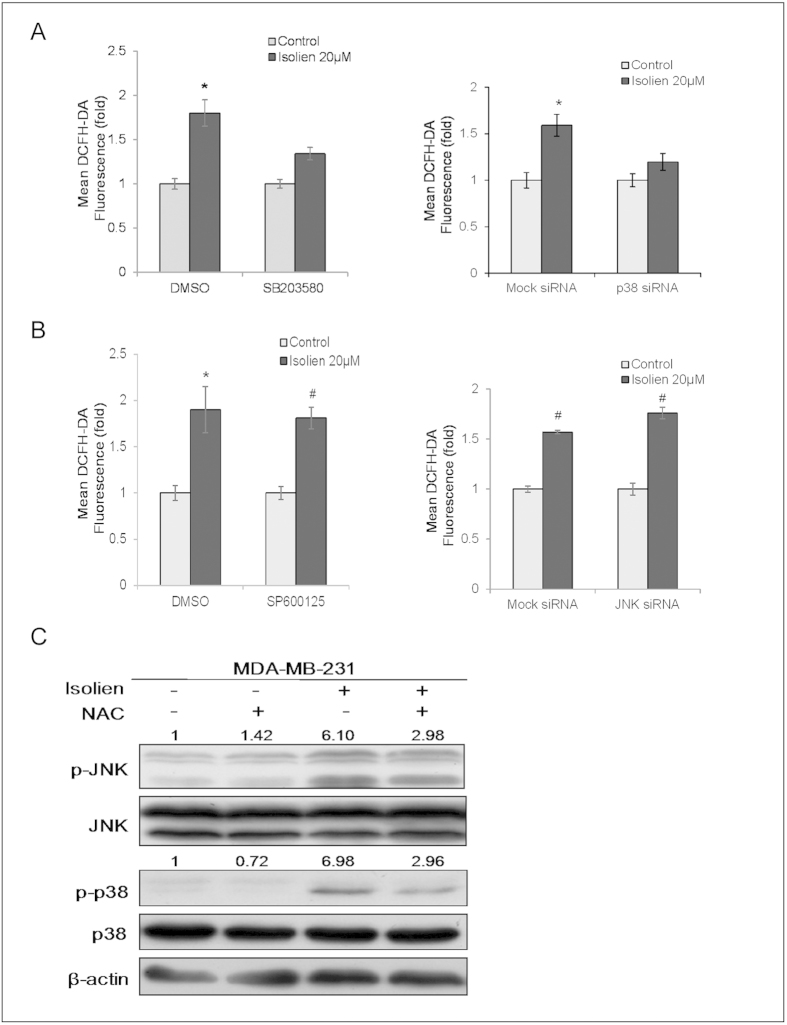
Effect of p38 MAPK and JNK inhibition on isoliensinine-induced ROS generation. **A**,**B**, MDA-MB-231 cells were pretreated with inhibitors (10 μM SB203580 or 10 μM SP600125) for 1 h, or transfected with specific siRNAs, and then 20 μM isoliensinine was added for additional 24 h. ROS production was measured using DCFH-DA by flow cytometry. Data presented as means ± S.D. are representative of three independent experiments. *P < 0.05, #P < 0.01, when compared with the vehical control. **C**, cells were pretreated with 10 mM NAC for 1 h, and then followed by 20 μM isoliensinine for 24 h. Cell lysates were prepared and subjected to western blotting for the phosphorylation levels of p38 and JNK. β-actin was used as a loading control. For p-JNK1 and p-p38, band intensities were normalized to JNK1 and p38, respectively. Data are expressed as a fold change relative to the control. The full-length blots are included in the supplementary information (Figure S10).

Since isoliensinine induced ROS generation and activation of p38 MAPK and JNK, we next examined whether p38 MAPK and JNK are involved in isoliensinine-induced ROS generation in MDA-MB-231 cells. As shown in Fig. 6A, pretreatment with p38 MAPK inhibitor SB203580 or transfection with p38 specific siRNA could significantly attenuate the increase of ROS production by isoliensinine. However, inhibition of JNK by either pretreatment with inhibitor SP600125 or specific siRNA transfection did not significantly suppress isoliensinine-induced ROS generation (Fig. 6B). These results indicated that the activation of p38 MAPK, but not that of JNK, contributed to ROS generation induced by isoliensinine.

Because previous studies demonstrated that MAPK pathways could be activated by ROS^21^,^22^, we further evaluated the effect of NAC on the activation of p38 MAPK and JNK induced by isoliensinine. As shown in Fig. 6C, the levels of activated p38 MAPK and JNK in response to isoliensinine could be partially inhibited by NAC, suggesting that ROS also mediates the activation of p38 MAPK and JNK induced by isoliensinine. Taken together, these results indicated that p38 MAPK pathway and ROS generation may reinforce each other in driving apoptosis of MDA-MB-231 cells treated with isoliensinine.

## Discussion

Isoliensinine and its analogue neferine and liensinine have been reported as potential anti-cancer agents in many cancer cells^5^,^6^,^8^. In this study we firstly evaluated the growth-inhibitory effects of isoliensinine, liensinine and neferine on MDA-MB-231 cells. Although isoliensinine, liensinine and neferine resemble each other in structure, these alkaloids exhibited different anti-proliferation effect on MDA-MB-231 cells. Isoliensinine possessed the most potent cytotoxic effect, and liensinine showed the least cytotoxicity. We found that isoliensinine could trigger a significant increase in apoptosis of triple-negative breast cancer cells in a p38 MAPK and JNK-dependent manner. Importantly, ROS level was selectively induced by isoliensinine in triple-negative breast cancer cells, but not in MCF-10A cells. The increased ROS production mediated the pro-apoptotic effect of isoliensinine on cancer cells. These findings indicate that isoliensinine may possess selective cytotoxic effect on cancer cells.

Evidence has suggested that some natural compounds, such as curcumin^23^ and resveratrol^24^, behave either as anti-oxidant or pro-oxidant depending on the concentration applied and the target cells. Neferine, an analogue of isoliensinine, inhibited high glucose-induced apoptosis in endothelial cells by blocking ROS generation^17^. Neferine also could induce mitochondrial-mediated ROS generation in triggering apoptosis in HepG2 cells^6^. Isoliensinine showed a significant inhibitory effect on bleomycin-induced pulmonary fibrosis in mice, probably due to its anti-oxidant and anti-inflammatory activities^16^. Here, we found that while isoliensinine had a potent cytotoxic effect on breast cancer cells, it exhibited a minimal effect on normal human breast epithelial cells MCF-10A at low concentrations (Fig. 1C). Meanwhile, isoliensinine significantly increased ROS production in triple-negative breast cancer cells, but failed to do so in MCF-10A cells. ROS scavenger NAC could significantly attenuate apoptosis induced by isoliensinine in MDA-MB-231 cells. Cancer cells usually have higher ROS level than normal cells and are less tolerable to further ROS insults. Other natural product, piperlongumine, has been reported to selectively increase ROS levels and apoptotic cell death in cancer cells but not in normal cells^19^,^25^. Lanperisone was also shown to selectively kill K-ras mutant cells by inducing oxidative stress^26^. More study is needed to determine why isoliensinine selectively induces ROS in triple-negative breast cancer cells.

Known as stress activated protein kinases, p38 MAPK and JNK are activated by various stress stimuli. It has been widely established that activation of p38 MAPK and JNK plays a critical role in natural compounds-induced apoptosis^9^,^21^. Consistent with these reports, isoliensinine induced significant elevation in the phosphorylation of p38 MAPK and JNK in a time-dependent manner in triple-negative breast cancer cells. In addition, inhibition of p38 MAPK and JNK, by SB203580 and SP600125, respectively, reduced isoliensinine-induced cleavage of PARP-1. Depletion of p38 or JNK by siRNA produced similar results, suggesting that p38 MAPK and JNK were involved in isoliensinine-induced apoptosis. A number of studies reported that pro-apoptotic p38 MAPK and JNK were activated by ROS^6^,^12^,^15^,^21^. Indeed, we found that apoptosis and p38 MAPK and JNK activation induced by isoliensinine were significantly attenuated by NAC. On the other hand, SB203580 and p38 specific siRNA could also markedly reduce isoliensinine-induced ROS generation in MDA-MB-231 cells, which is consistent with the reports that ROS generation was mediated by activation of p38 MAPK^13^,^14^. Therefore, p38 MAPK pathway and ROS may augment each other during isoliensinine-induced apoptosis in triple-negative breast cancer cells. The mechanism by which isoliensinine activates p38 MAPK and increases ROS production in triple-negative breast cancer cells may bear some analogy to a recently described JNK-NADPH oxidase-ROS self-driven signal circuit in hepatic carcinoma cells^22^.

In summary, isoliensinine could efficiently induce apoptosis in triple-negative breast cancer cells. Its cytotoxic effect was achieved by inducing oxidative stress and by activating p38 and JNK pathways. Therefore, our findings suggest that isoliensinine may serve as a potential anti-cancer agent for TNBCs.

## Methods

### Cell cultures

Human breast cancer cells MDA-MB-231, MDA-MB-436, MDA-MB-468, MCF-7 and normal human breast epithelial cells MCF-10A were obtained from the American Type Culture Collection (Manassas, VA). MDA-MB-231, MDA-MB-436, MDA-MB-468, MCF-7 cells were cultured in DMEM medium supplemented with 10% FBS (Gibco, Invitrogen), 100 U/ml penicillin and 100 μg/ml streptomycin. MCF-10A cells were grown in DMEM/F12 Medium (Gibco, Invitrogen), containing 5% horse serum (Gibco, Invitrogen), 20 ng/ml EGF, 0.5 mg/ml hydrocortisone, 100 ng/ml Cholera Toxin, 10 μg/ml insulin, 100 U/ml penicillin and 100 μg/ml streptomycin. All cells were cultured at 37 °C with 5% CO_2_.

### Reagents

Isoliensinine, liensinine and neferine (98% by HPLC, Tianhaoyuan Biotech Co., Ltd., Tianjin, China) were dissolved in DMSO (maximum concentration, 20 mg/ml), respectively. DMSO was also applied to controls. The antibodies against p21 (sc-6246), cyclin E (sc-25303), Bcl-2 (sc-7382), Bax (sc-7480), and β-actin (sc-69879) were acquired from Santa Cruz Biotechnology. The antibodies against Caspase-3 (9662), PARP-1 (9532), phospho-p38 MAPK (9215), p38 MAPK (9212), JNK1/2 (9258), and phospho-JNK1/2 (4668) were from Cell Signaling Technology. NAC, SB203580 and SP600125 were purchased from Beyotime Institute of Biotechnology (China).

### Cell proliferation assay

Cell proliferation was measured by the CCK-8 assay kit (Beyotime, China). Briefly, cells were seeded in 96-well culture plates the day before isoliensinine treatment. After incubation, 10 μl of CCK-8 reagent was added to each well and the absorbance was measured at 450 nm 2 h later. All experiments were repeated at least three times.

### Colony formation assay

Breast cancer cells were plated on 6-well plates at a density of 1 × 10^3^ cells/well and treated with isoliensinine at different concentrations. Media were changed after 48 h of incubation. Colonies were scored 2 weeks later. Colonies were fixed and stained with 0.1% crystal violet in 10% ethanol and counted.

### Cell cycle analysis

Cell cycle analysis was performed as previously described^27^. After being treated with isoliensinine for the indicated times, the adherent cells were washed once with PBS, trypsinized, and collected by centrifugation at 400 × g for 5 min. The cells (10^6^ cells per sample) were fixed in 4 ml of cold 70% ethanol at –20 °C overnight. After centrifugation at 1000 × g for 10 min, cell pellets were incubated with 0.5 ml of PBS containing 100 μg/ml RNase (Invitrogen) and 5 μg/ml propidium iodide (Sigma-Aldrich) at room temperature for 30 min. Cell cycle distribution was analyzed by measuring DNA content using flow cytometry.

### Edu proliferation assay

MDA-MB-231 cells plated in 96-well plate were treated with isoliensinine at the indicated concentrations for 48 h, cell proliferation was detected using the incorporation of 5-ethynyl-2′-deoxyuridine (EdU) with the EdU cell proliferation assay kit (Guangzhou RiboBio Co., Ltd. Guangzhou, China). Briefly, the cells were incubated with 50 μM EdU for 2 h before fixation, permeabilization and EdU staining according to the manufacturer’s protocol. The nuclei were stained with Hoechst33342. The proportion of EdU positive cells was determined by fluorescence microscopy.

### Annexin V apoptosis assay

Apoptotic cells were identified by Annexin V/Dead Cell Apoptosis Kit (Invitrogen). Briefly, MDA-MB-231 cells were treated with different concentrations of isoliensinine for 48 h and with 20 μM isoliensinine for 24, 48 and 72 h. Thereafter, cells were harvested and washed twice with ice-cold PBS. Cells were resuspended in annexin-binding buffer and incubated at room temperature for 15 mins in the dark after 5 μl Annexin V-FITC and 1 μl PI additions. FITC fluorescence was analyzed by flow cytometry.

### Measurement of ROS

ROS generation was measured using oxidation sensitive fluorescent probe (DCFH-DA) according to the manufacturer’s protocols (Beyotime, China). Breast cancer cells were treated with isoliensinine in the absence or presence of NAC, SB203580 and SP600125 for the indicated times. MCF-10A cells were exposed to 20 μM isoliensinine for 48 h. After the incubation, the cells were harvested and then stained with 10 μM DCFH-DA probe at 37 °C for 20 min. Cells were washed three times with PBS, and the induction of ROS was examined by flow cytometry. In all experiments, 20,000 viable cells were analyzed.

### RNA interference

All small interfering RNAs (siRNAs) were purchased from GenePharma Co. (Shanghai GenePharmaCo., China). The siRNA targeting human alpha p38 MAPK (5′-GGGCAGAUCUGAACAACAU-3′)and beta p38 MAPK(5′-GAGCGACGAGCACGUUCAA-3′) were applied as a mixture at a total final concentration of 80 nM. The siRNA targeting human JNK1 (5′-GCCCAGUAAUAUAGUAGUA-3′) and JNK2 (5′-GUUGCAGUCAAGAAACUAA-3′) were used as a mixture at a total final concentration of 80 nM. A non-silencing scramble RNA duplex was used as the negative control (5′-UUCUCCGAACGUGUCACGU-3′). MDA-MB-231 cells were transfected with siRNAs using Lipofectamine^2000^ (Invitrogen, CA) as described previously^5^. Briefly, 2 × 10^5^ MDA-MB-231 cells were seeded in 60 mm dishes in antibiotic-free medium. The next day, siRNAs were introduced into the cells using lipofectamine^2000^ transfection reagent according to the manufacturer’s protocols. MDA-MB-231 cells were replated in 60 mm dishes at a concentration of 8 × 10^4^ cells/ml 24 h after transfection. After the incubation in medium containing isoliensinine for the indicated time, cells were then harvested for immunoblotting.

### Western blotting

Cells were harvested and lysed on ice for 20 min in lysis buffer (Beyotime, China). The protein concentration was determined by the BCA assay kit (Beyotime, China). 30–50 μg protein samples were separated by SDS-PAGE (6–12%) and electro-transferred onto PVDF membrane. The membrane was blocked with 5% skim milk and incubated with specific primary antibodies at 4 °C for overnight. Proteins of interest were detected with appropriate horseradish peroxidase-conjugated secondary antibodies and developed using ECL kit (Thermo). The protein levels were normalized by β-actin.

### Statistical analysis

All data were expressed as mean ± standard derivation (SD) of three independent experiments. A statistical significance test was performed with analysis of variance followed by one-way ANOVA test for experiments consisting of more than three groups. Student’s t test was used to analyze all other data, with P < 0.05 considered as the level of significance.

## Additional Information

**How to cite this article**: Zhang, X. *et al.* Isoliensinine induces apoptosis in triple-negative human breast cancer cells through ROS generation and p38 MAPK/JNK activation. *Sci. Rep.* **5**, 12579; doi: 10.1038/srep12579 (2015).

## Supplementary Material

Supplementary Information
