# Supplementary material for: Isoliensinine induces apoptosis in triple-negative human breast cancer cells through ROS generation and p38 MAPK/JNK activation
Source: Sci Rep. 2015 Jul 29;5:12579. doi: 10.1038/srep12579 (PMC4518223; doi:10.1038/srep12579)
Supplement: Supplementary Information [file srep12579-s1.pdf]

Supplementary Information

Isoliensinine induces apoptosis in triple-negative human breast cancer cells through ROS generation and p38 MAPK/JNK activation

Xiyu Zhang[1*], Xiyao Wang[1], Tingting Wu[1], Boxuan Li[1], Tianqi Liu[1], Rong Wang[2], Qiao Liu[1], Zhaojian Liu[3], Yaoqin Gong[1]and Changshun Shao[1*]

[1]Key Laboratory of Experimental Teratology, Ministry of Education andDepartment of Molecular Medicine and Genetics,Shandong University School of Medicine, Jinan, Shandong 250012, China.

[2]Department of Physiology, Shandong University School of Medicine, Jinan, Shandong 250012, China.

[3]Department of Cell Biology, Shandong University School of Medicine, Jinan, Shandong 250012, China.

[*]Corresponding Authors

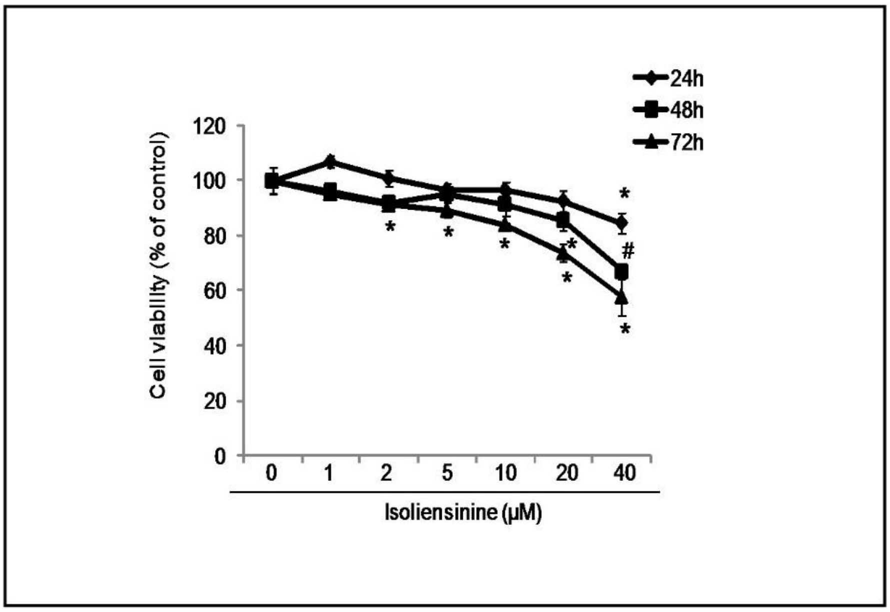

Figure S1. MCF-10A cells were exposed to isoliensinine (1-40μM) or vehicle control (0.1% DMSO) for 24h, 48h and 72h. Cell viability was measured by CCK-8 assay. The experiments were performed in triplicate. Data presented as means ± S.D. are representative of three independent experiments. *P<0.05, ＃P<0.01, when compared with control group.

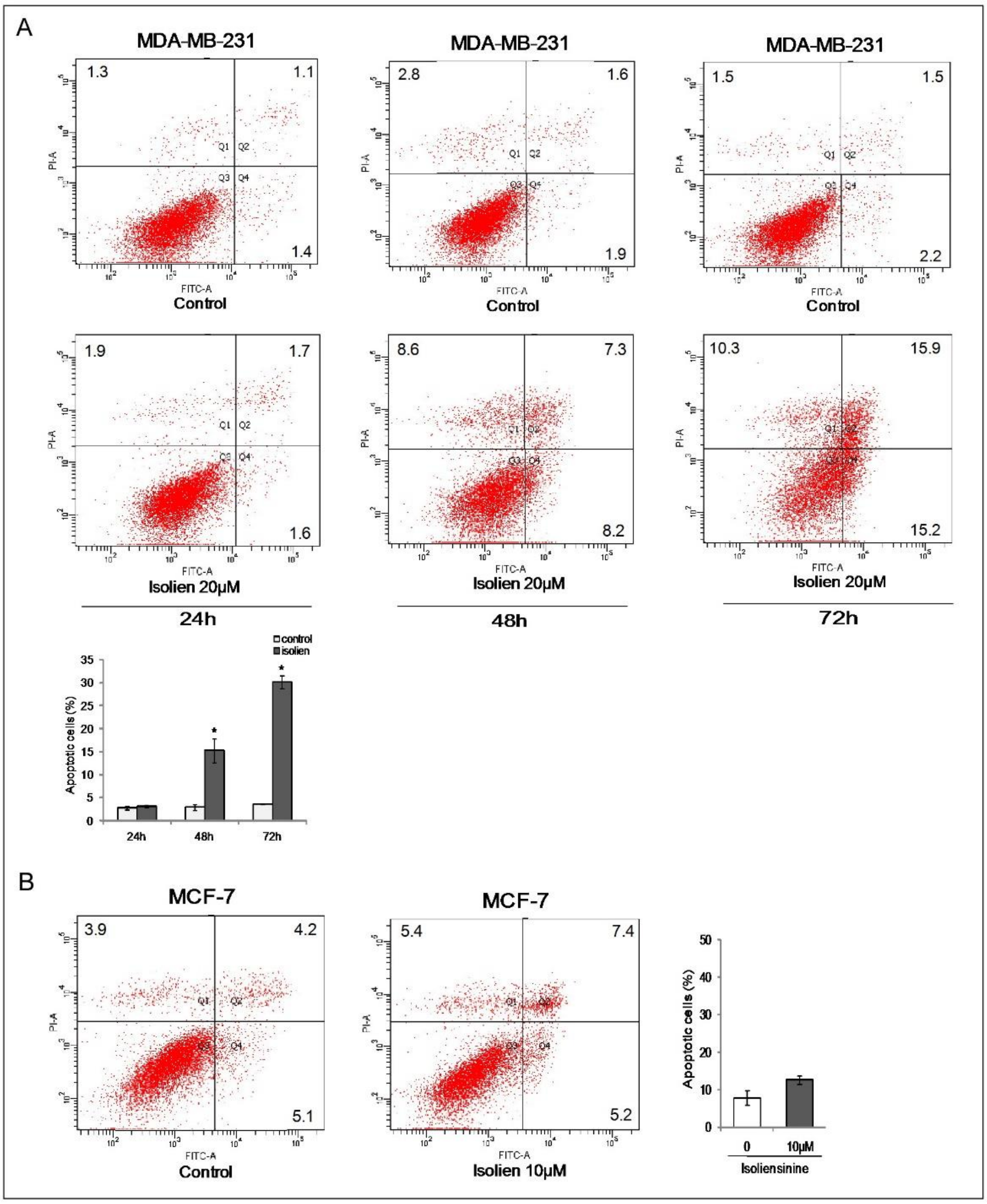

Figure S2. A, Effect of isoliensinine treatment on apoptosis in MDA-MB-231 cells. MDA-MB-231 Cells were treated with 20μM isoliensinine for the indicated times (24h, 48h and 72h). B, Effect of isoliensinine treatment on apoptosis in MCF-7 cells. MCF-7 cells were incubated with 20μM isoliensinine for 48h. Apoptosis was analyzed as in Figure 3A. The percentage of Annexin V-positive population indicates apoptosis induction at the indicated lengths of time. Results shown are representative of three independent experiments. *P<0.05, ＃P<0.01, when compared with control group.

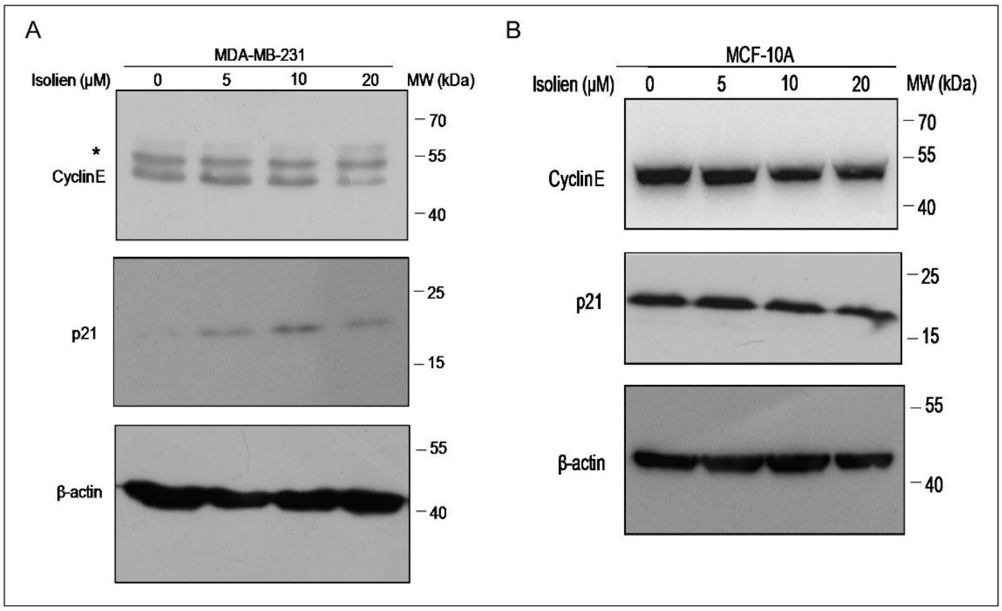

Figure S3. Effect of isoliensinine on cell cycle distribution in MDA-MB-231 cells (A) and normal human breast epithelial cells MCF-10A (B). The cropped blots are used in the main figure (Figure 2D, 2G).

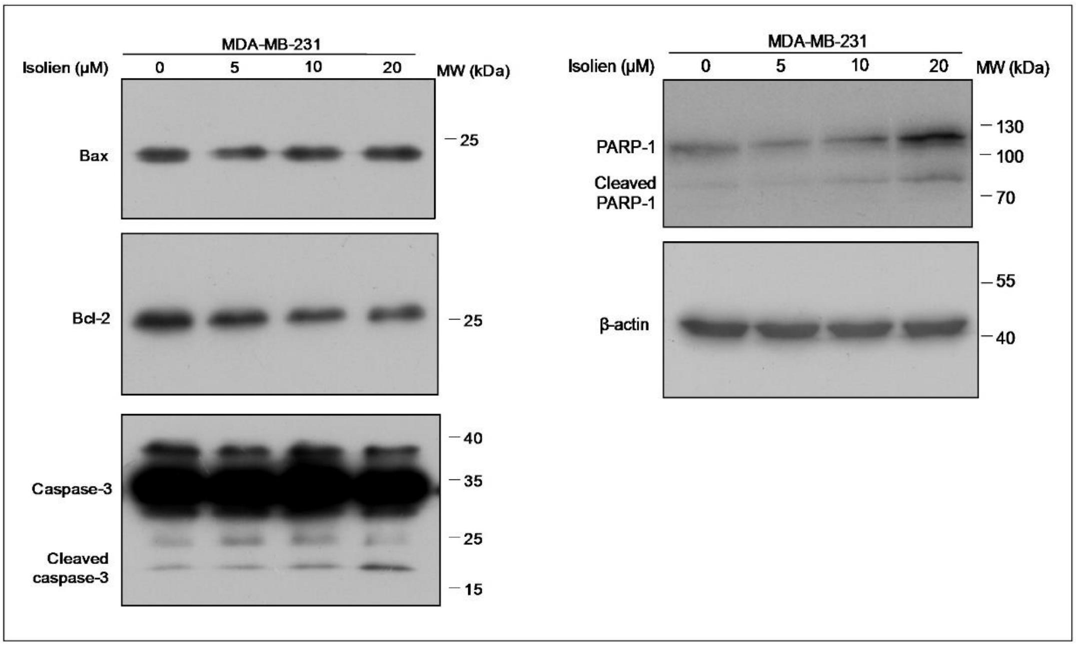

Figure S4. Pro-apoptotic effect of isoliensinine on MDA-MB-231 cells. The cropped blots are used in the main figure (Figure 3B).

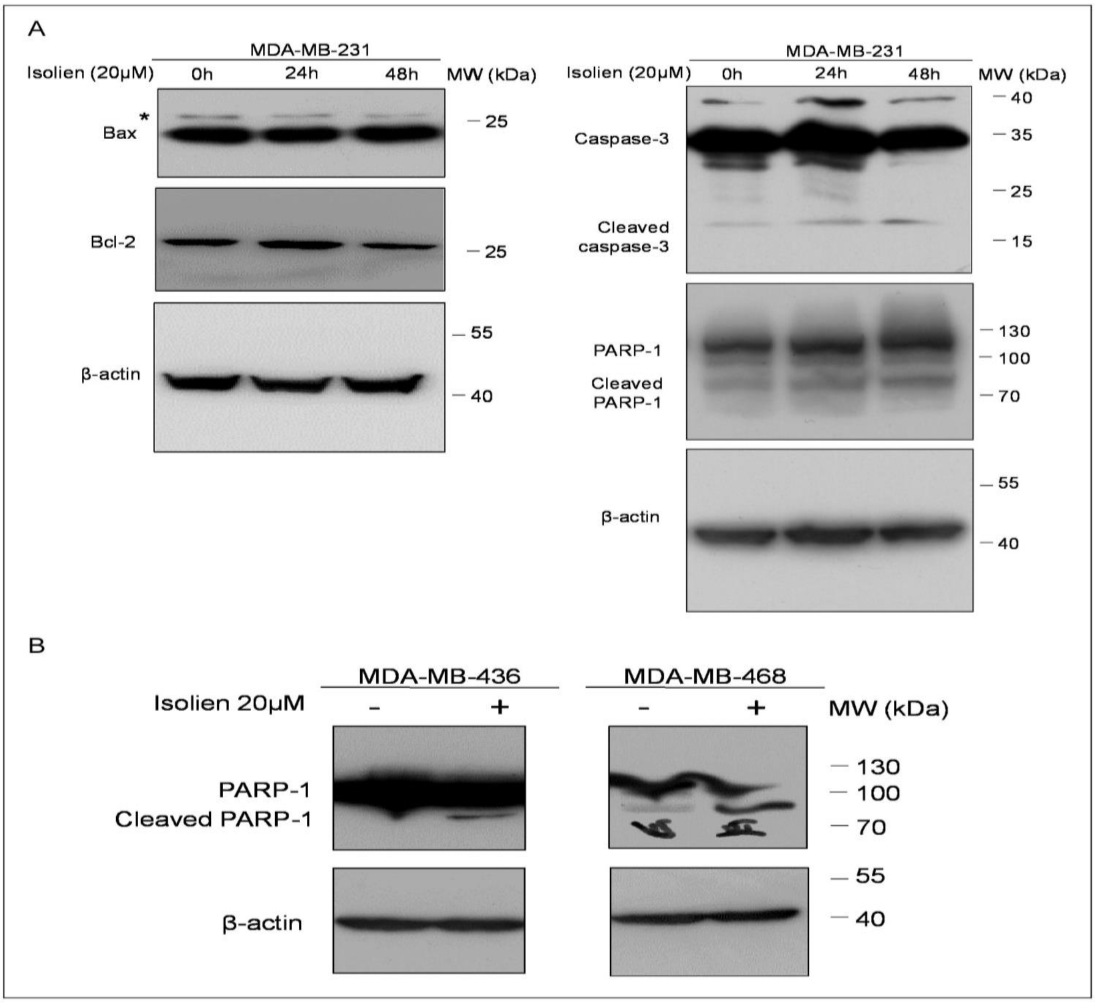

Figure S5. Pro-apoptotic effect of isoliensinine on MDA-MB-231 cells (A), MDA-MB-436 and MDA-MB-468 cells (B). The cropped blots are used in the main figure (Figure 3C and 3D).

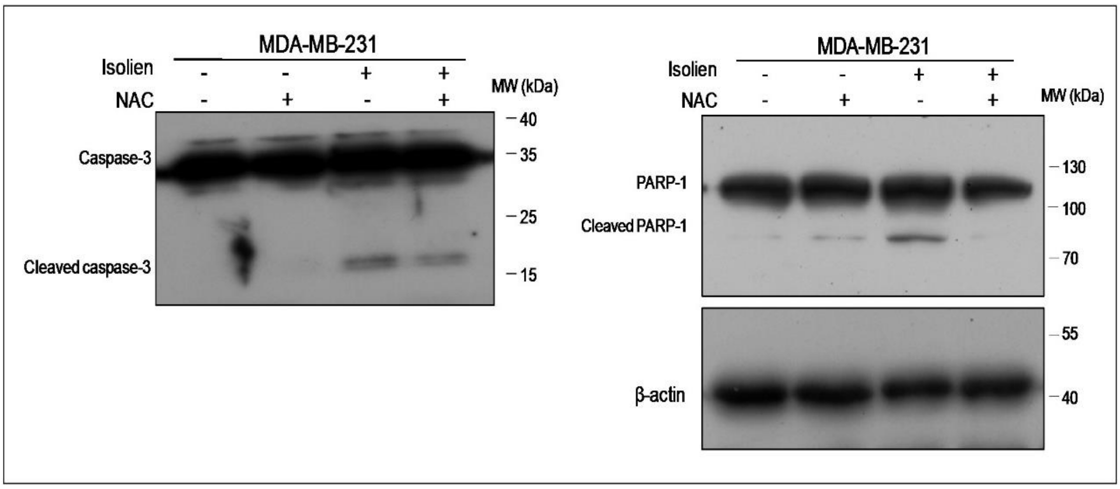

Figure S6. Effect of isoliensinine on expression of apoptosis-related proteins in the absence or presence of NAC. The cropped blots are used in the main figure (Figure 4G).

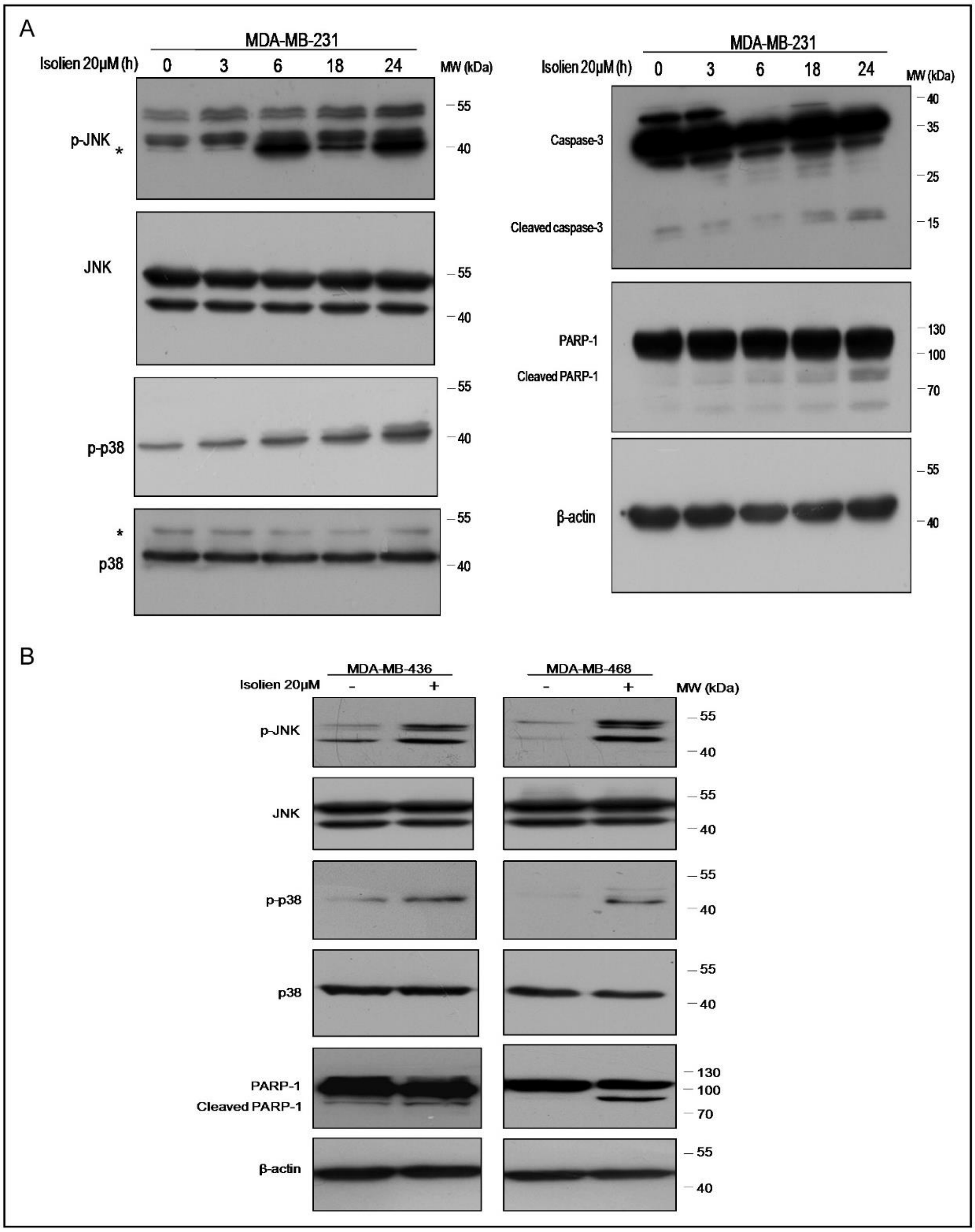

Figure S7. p38 MAPK and JNK pathways mediate isoliensinine-induced apoptosis in MDA-MB-231 (A), MDA-MB-436 and MDA-MB-468 cells (B). The cropped blots are used in the main figure (Figure 5A and 5B).

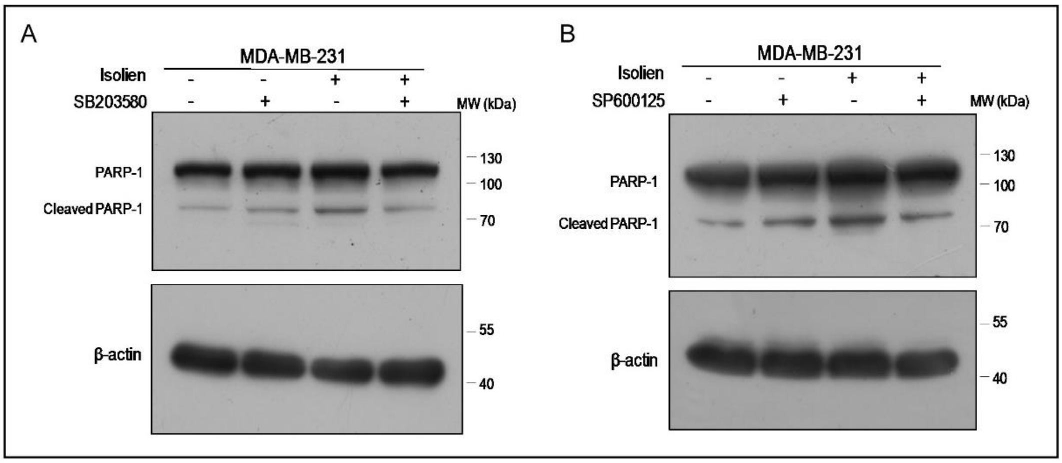

Figure S8. p38 MAPK and JNK pathways mediate isoliensinine-induced apoptosis in MDA-MB-231 cells. MDA-MB-231 cells were treated with 20μM isoliensinine alone or in combination with 10μM SB203580 (A) or 10μM SP600125 (B) for 24h, respectively. The cropped blots are used in the main figure (Figure 5C and 5D).

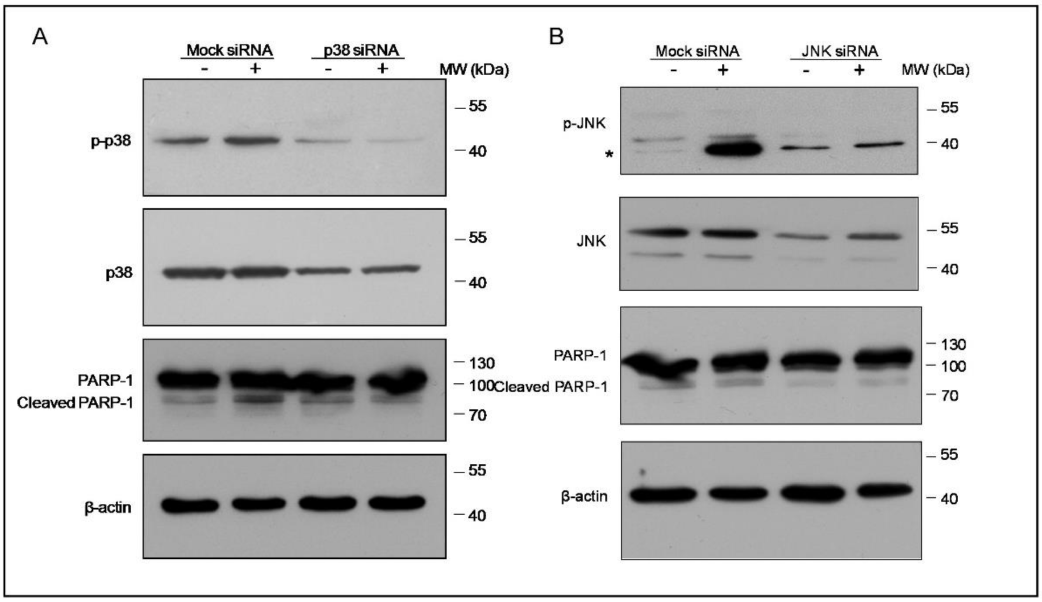

Figure S9. p38 MAPK and JNK pathways mediate isoliensinine-induced apoptosis in MDA-MB-231 cells. MDA-MB-231 cells were transfected with mock siRNA, p38 siRNA (A) or JNK siRNA (B), and incubated with 20μM isoliensinine for 24h. The cropped blots are used in the main figure (Figure 5E and 5F).

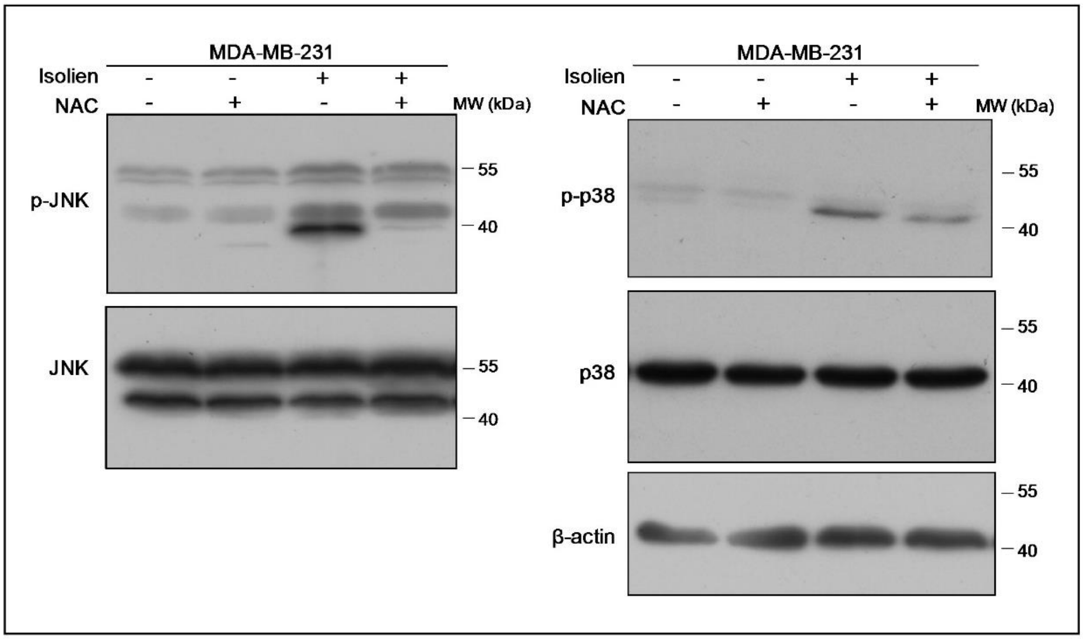

Figure S10. Effect of NAC on the activation of p38 MAPK and JNK induced by isoliensinine.

The cropped blots are used in the main figure (Figure 6C).
